# The Association between Dietary Energy Density and Type 2 Diabetes in Europe: Results from the EPIC-InterAct Study

**DOI:** 10.1371/journal.pone.0059947

**Published:** 2013-05-16

**Authors:** The InterAct Consortium

**Affiliations:** 1 Membership of the InterAct Consortium is provided in the Acknowledgments.; University of Colorado Denver, United States of America

## Abstract

**Background:**

Observational studies implicate higher dietary energy density (DED) as a potential risk factor for weight gain and obesity. It has been hypothesized that DED may also be associated with risk of type 2 diabetes (T2D), but limited evidence exists. Therefore, we investigated the association between DED and risk of T2D in a large prospective study with heterogeneity of dietary intake.

**Methodology/Principal Findings:**

A case-cohort study was nested within the European Prospective Investigation into Cancer (EPIC) study of 340,234 participants contributing 3.99 million person years of follow-up, identifying 12,403 incident diabetes cases and a random subcohort of 16,835 individuals from 8 European countries. DED was calculated as energy (kcal) from foods (except beverages) divided by the weight (gram) of foods estimated from dietary questionnaires. Prentice-weighted Cox proportional hazard regression models were fitted by country. Risk estimates were pooled by random effects meta-analysis and heterogeneity was evaluated. Estimated mean (sd) DED was 1.5 (0.3) kcal/g among cases and subcohort members, varying across countries (range 1.4–1.7 kcal/g). After adjustment for age, sex, smoking, physical activity, alcohol intake, energy intake from beverages and misreporting of dietary intake, no association was observed between DED and T2D (HR 1.02 (95% CI: 0.93–1.13), which was consistent across countries (*I* ^2^ = 2.9%).

**Conclusions/Significance:**

In this large European case-cohort study no association between DED of solid and semi-solid foods and risk of T2D was observed. However, despite the fact that there currently is no conclusive evidence for an association between DED and T2DM risk, choosing low energy dense foods should be promoted as they support current WHO recommendations to prevent chronic diseases.

## Introduction

Over the past decades the prevalence of type 2 diabetes (T2D) has increased dramatically, and it is estimated that the global prevalence of diabetes will continue to rise, with an estimated 489 million cases in the year 2030, increased from an estimated 285 million in 2010 [1]. Major non-genetic risk factors for T2D such as obesity, smoking, physical inactivity and diets high in (saturated) fat and low in dietary fiber are potentially modifiable [2]. Targeting those risk factors by lifestyle intervention among high risk individuals has shown to be effective in the prevention of T2D [3], [4], [5].

The energy density of foods or diets is defined as the amount of available energy per unit weight of foods or meals (kJ/g or kcal/g) [6]. Experimental data suggest that people tend to eat a similar volume of food to feel satiated, and accordingly consumption of energy-dense foods could cause passive over-eating in terms of energy [7]. Several observational studies have observed a positive association between dietary energy density (DED) and subsequent weight or waist circumference gain [8], [9], [10].

As obesity is an important and well known risk factor for T2D [2], it is likely that consumption of energy dense foods might be associated with an increased risk of T2D. Besides this indirect effect, a direct effect of DED on T2D risk can also be hypothesized. High energy dense diets are associated with low diet quality characterized by, amongst other features, a higher intake of saturated fat and a higher glycemic load [10], [11]. Both of these dietary factors have been associated with the development of insulin resistance [12], [13]. So far, there is limited epidemiological evidence for an association between DED and risk of T2D [14] or related metabolic traits [15], [16].

Our aim was to investigate the association between DED and risk of T2D. A secondary objective was to perform stratified analysis of the association between DED and T2D risk by BMI status and energy under-reporting. We had the opportunity to do this in the EPIC-InterAct project which assembled a large case-cohort study in European populations with substantial diversity of dietary intakes.

## Materials and Methods

### Ethics statement

This study complied with the Declaration of Helsinki. The Internal Review Board of the International Agency for Research on Cancer and the Institutional Review board of all centers, i.e., France, Heidelberg, Potsdam, Copenhagen, Aarhus, Asturias, Granada, San Sebastian, Murcia, Navarro, Cambridge, Oxford, Imperial, Florence, Milan, Ragusa, Turin, Naples, Bilthoven, Utrecht, Malmö, and Umeä, approved the EPIC study. Written consent was obtained from each EPIC participant at enrolment into the study.

### Study design

A case-cohort study was nested within the European Prospective Investigation into Cancer and Nutrition (EPIC) [17] as part of the InterAct Project, hereafter referred to as the EPIC-InterAct Study. With the exception of Norway and Greece, all other eight EPIC countries (France, Italy, Spain, UK, the Netherlands, Germany, Sweden and Denmark) participated in the EPIC-InterAct study.

An extensive description of the study design and cohort has been published elsewhere [18]. In brief, the EPIC-InterAct case-cohort study consists of 12,403 verified incident T2D cases recruited between 1991 and 1997. In addition, a center stratified random subcohort of 16,835 individuals was selected from the baseline cohort (n = 340,234; 3.99 million person years). After exclusion of 548 individuals with prevalent diabetes and 133 with unknown diabetes status, 16,154 subcohort individuals were included, of whom 778 had developed T2D between 1991 and 2007. EPIC participants without stored blood or reported diabetes status were not eligible for inclusion in the study. For the present study, participants with missing information on diet and therefore DED (n = 117) and on covariates (smoking status, physical activity, BMI, energy intake from beverages; n = 364) were excluded to allow a complete case analysis. In addition, participants who did not complete the FFQ adequately, identified as the top and bottom 1% of the ratio for energy intake to estimated basal metabolic rate (EI/BMR), were excluded (n = 619). In total, 11,734 incident T2D cases and 15,434 subcohort individuals (5,825 men and 9,609 women), of whom 733 had developed incident T2D were eligible for analysis. An overlap between the case set and the sub-cohort is a design feature of a case-cohort study [19].

### Assessment of T2D

A pragmatic, high sensitivity approach was used for case ascertainment with the aim of identifying all potential incident T2D cases and excluding all individuals with prevalent diabetes [18]. Prevalent cases were identified on the basis of baseline self-report of a history of diabetes, doctor diagnosed diabetes, anti-diabetic drug use or evidence of diabetes, where the date of diagnosis preceded recruitment. Ascertainment of incident diabetes involved a review of the existing EPIC datasets at each center using multiple sources of evidence including self-report (self-reported history of diabetes, doctor diagnosed diabetes, anti diabetic drug use), linkage to primary care registers, secondary care registers, medication use (drug registers), hospital admissions and mortality data. Cases in Denmark and Sweden were not ascertained by self-report, but identified via local and national diabetes and pharmaceutical registers and hence all ascertained cases were considered to be verified. To increase the specificity of the case definition for centers other than those from Denmark and Sweden, identified cases were verified with further evidence, including reviewing individual medical records in some centers.

The date of diagnosis for incident cases was set as either the date of diagnosis reported by the doctor, the earliest date that diabetes was recorded in medical records, the date of inclusion into the diabetes registry, the date reported by the participant, or the date of the questionnaire in which diabetes was first reported. If the date of diagnosis could not be ascertained from any of the sources listed above, the midpoint between recruitment and censoring was used. Follow up was censored at the date of diagnosis, 31^st^ of December 2007 or the date of death, whichever occurred first.

### Dietary assessment

The assessment of diet was undertaken using a self- or interviewer-administered dietary questionnaire which was developed and validated within each country to estimate the usual individual food intakes of study participants [17], [20], [21]. In a previous study, DED measured by the Dutch FFQ was validated against DED derived from the weighted average of multiple 24-hour recalls in a subset of this study population [10]. Results indicated a good validity of the DED values measured by this FFQ (Spearman correlation coefficients: 0.64 in men and 0.56 in women).

DED was calculated by dividing daily energy intake (kcal) from foods (solid foods and semi-solid or liquid foods such as soups) by the reported weights (g) of these foods. It was decided *a priori* to exclude caloric and non-caloric beverages (including water, tea, coffee, juices, soft drinks, alcoholic drinks and milk) from the DED calculation. The main reason was that DED calculations based on the inclusion of beverages were associated with higher day to day variation within individuals [22]. This may diminish associations when examining health outcomes [22]. Also, beverages may add more weight than energy to diets, thereby lowering individual DED values disproportionately [22].

To be able to identify under-reporters and over-reporters of diet, the ratio of energy intake versus basal metabolic rate (EI/BMR) was calculated and compared with Goldberg cut-off values [23], [24]. BMR was estimated using the Schofield equation [25]. Participants with a ratio of EI/BMR below 1.14 were classified as under-reporters, those with a ratio of EI/BMR above 2.1 were defined as over-reporters, whereas all other participants were defined as plausible reporters of diet [26].

### Other measurements

At baseline, information on lifestyle was collected via self-administered questionnaires included amongst others age, sex, educational level, physical activity, smoking and menopausal status. Smoking status was divided into current, former or never smoking. Women were classified as pre-, peri-, post- or surgical postmenopausal. Education level was indicated as the highest level of school achieved and participants were classified into either primary school or less, technical-professional school, secondary school, university or higher. Physical activity level was assessed using a brief validated questionnaire covering occupational and recreational activities and participants were classified as either inactive, moderately inactive, moderately active or active [27].

Body weight, height and waist circumference were measured at baseline by trained technicians using standard study protocols as previously described [28]. BMI was calculated as body weight in kilograms divided by the square of the height in meters. Normal weight, overweight and obesity were defined as a BMI<25 kg/m^2^, between 25 kg/m^2^ and 30 kg/m^2^ and ≥30 kg/m^2^, respectively.

### Statistical analyses

Characteristics of the subcohort participants at baseline were examined by quintiles of DED which were derived from the DED distribution in the overall subcohort. Means and standard deviations were presented for describing continuous data, except when the data were not normally distributed, in which case medians and interquartile ranges were given. For categorical variables percentages were presented. The DED variable had a normal distribution. Stepwise linear regression analysis was performed (significance level entry = 0.15, significance level stay = 0.15) to investigate the contribution of food groups and nutrients to the inter-individual variation in DED in two separate models. All standard derived main food groups [21], except beverages, were entered into the regression model: potatoes, vegetables, legumes, fruits, dairy products, nuts and seeds, cereals, meat, fish, eggs, fats, sugar and confectionery, cakes and biscuits, condiments and sauces, soups, and miscellaneous. Seven macronutrients: saturated fatty acids, monounsaturated fatty acids, polyunsaturated fatty acids, polysaccharides, mono-&disaccharide carbohydrates, animal protein and plant protein were also entered in a regression model.

Since we used data from a subcohort instead of the full cohort, a weighted Cox regression suitable to case-cohort designs was applied to derive the hazards ratios (HRs) for incident T2D [19]. Prentice weights were used i.e. all subcohort members (cases and non-cases) are weighted equally and cases outside the subcohort are not weighted before failure [29]. At failure these cases have the same weight as the subcohort members (also called the unweighted method). Models were fitted separately for each country with time as the underlying timescale. HRs were calculated for incident T2D per 1 kcal/g increase in DED and quintiles of DED. Random effects meta-analysis was performed to pool hazard ratios across countries and evaluate heterogeneity (*I^2^*) between countries. In addition, forest plots were generated.

Age, sex, center, educational level, smoking, physical activity, alcohol (g/day), energy intake from beverages (kcal/day), family history of diabetes and misreporting of diet were considered as potential confounding variables. These variables were added one by one to the crude model. Variables that changed the β-coefficient for DED by ten percent or more were added to the multivariable models. The first model included sex and age. Additional adjustments were subsequently made for known risk factors of diabetes: smoking status, physical activity, consumption of alcohol, energy intake from beverages (model 2). In model 3 further adjustments were made for misreporting of diet (under-, plausible and over-reporters). Additional adjustment for menopausal status was made in women only. In case of a statistically significant association between DED and incident T2D (model 3), potential mechanisms underlying the association were explored by addition of BMI, waist circumference and several dietary factors (energy intake, fiber, total fat, fruits and vegetables) one by one.

Potential interactions of DED with sex, age (2 groups based on median of 54.1 years), BMI, waist circumference, physical activity and misreporting of diet were investigated by the inclusion of interaction terms to model 3. Under-reporting of diet is a known phenomenon in epidemiological studies on diet-disease relationships, especially among obese individuals [30]. To get more insight in this potential bias, characteristics of BMI status and misreporting of diet were explored by country. In addition, regression models were stratified by status of misreporting of diet (except for over reporters, due to small numbers) for normal weight, overweight and obese individuals separately. To be able to compare our results with previous related research by Wang et al^14^ within the EPIC NORFOLK cohort (which is also part of the EPIC-InterAct study) and to get more insight in methodological issues regarding DED calculation, a sensitivity analysis was performed based on DED calculations including all foods and all beverages except water.

Apart from the random-effect meta-analysis, which was conducted using STATA 11.0 (Stata-Corp, Texas, USA), all analyses were performed using SAS 9.2 (SAS, Institute, Cary, NC). P-values<0.05 were considered to be statistically significant.

## Results

Estimated mean DED was 1.5 kcal/g (SD 0.3) within the subcohort as well as among incident diabetes cases. The highest mean DED was observed in Germany (1.7 kcal/g) whereas the lowest DED was found in France, Spain and the UK (1.4 kcal/g). Participants with a higher DED were more often men and current smokers with a higher educational level and had a higher ratio of EI/BMR (**Table 1**). Furthermore, they were younger, had a lower BMI and waist circumference and less often reported having hypertension or hyperlipidemia. Although consuming a lower amount (total grams) of foods, participants with a higher DED had higher intake of total energy, total fat, dietary fiber and energy from beverages and a lower intake of fruit and vegetables. These differences in baseline characteristics were observed for both sexes (data not shown).

**Table 1:** Baseline characteristics of the subcohort (n = 15,434) across quintiles of dietary energy density: the EPIC-InterAct Study.

Characteristics	Overall *n = 15,434*	Q1*n = 3,035*	Q2*n = 3,189*	Q3*n = 3,134*	Q4*n = 3,059*	Q5*n = 3,017*
Energy density 1 (kcal/g)^2^	1.5 (0.3)	1.1 (0.1)	1.4 (0.1)	1.5 (0.04)	1.7 (0.1)	2.0 (0.2)
Age, years^2^	52.4 (9.1)	53.7 (8.9)	53.4 (8.9)	52.4 (9.2)	51.7 (8.9)	51.0 (9.4)
Sex (women)^3^	62.3	80.6	70.0	62.4	54.5	43.4
Education 3						
Primary or lower	40.9	47.8	43.1	39.5	37.1	36.9
Technical/professional	23.2	18.9	22.0	23.4	25.0	27.0
Secondary school	15.1	14.6	16.0	15.9	14.9	14.3
University degree or higher	20.7	18.7	18.9	21.3	23.0	21.8
Waist circumference, cm^2^						
Men	95.1 (10.0)	96.6 (10.4)	96.3 (9.7)	95.5 (9.5)	94.6 (9.9)	94.0 (10.3)
Women	81.2 (11.2)	82.3 (11.2)	81.6 (11.0)	80.7 (11.1)	80.8 (11.2)	79.7 (11.2)
Body mass index 2, kg/m^2^	26.0 (4.2)	26.5 (4.4)	26.2 (4.3)	25.9 (4.1)	25.9 (4.1)	25.6 (4.0)
Smoking 3						
Never	46.9	57.5	52.0	47.6	42.0	35.3
Former	27.2	26.7	25.3	28.2	28.3	27.6
Current	25.9	15.8	22.7	24.2	29.8	37.1
Physical activity index 3						
Inactive	23.7	27.0	24.5	22.8	20.6	23.5
Moderately inactive	33.7	33.2	34.3	33.9	34.1	32.9
Moderately active	22.7	21.1	21.5	23.1	24.7	22.9
Active	20.0	18.8	19.7	20.3	20.6	20.7
Family history diabetes, yes^3^	19.1	19.6	20.0	18.7	19.9	17.6
Hypertension, yes^3^	18.9	20.3	19.5	19.2	18.5	16.7
Hyperlipidemia, yes^3^	18.6	19.0	19.7	19.1	17.3	17.4
Menopausal status, postmenopausal^3^	47.5	52.6	51.4	47.7	40.1	40.1
Hormone replacement therapy, users^3^	14.9	14.2	16.1	14.9	14.9	14.0
**Dietaryintake** (per day)						
Energy, kcal^2^	2137 (634)	1783 (501)	2041 (566)	2157 (602)	2278 (615)	2431 (681)
Energy from beverages, kcal^4^	239 (143; 368)	184 (108; 289)	215 (132; 334)	246 (149; 369)	269 (167; 409)	294 (185; 440)
Total foods, g^2^	1236 (381)	1407 (412)	1309 (375)	1227 (360)	1169 (337)	1068 (326)
Fat, en%^2^	34.8 (5.9)	31.9 (6.0)	33.9 (5.2)	34.9 (5.3)	35.7 (5.3)	37.8 (5.9)
Fiber, g/1000 kcal^4^	10.6 (8.8; 12.7)	13.9 (12.0; 16.0)	11.5 (10.1; 13.1)	10.4 (9.0; 11.9)	9.6 (8.2; 11.1)	8.3 (7.0; 9.8)
Alcohol, g^4^	6.4 (0.8; 17.7)	2.7 (0.0; 11.2)	5.1 (0.6; 14.3)	6.8 (1.1; 19.4)	8.7 (1.7; 23.4)	8.4 (1.7; 23.3)
Fruit and vegetables, g^4^	376 (241;554)	628 (474; 831)	472 (346; 613)	373 (265; 509)	300 (212; 407)	203 (137; 292)
**Otherdietrelatedfactors**						
Glycemic load 4	126 (100;157)	106 (84;133)	122 (99;149)	127 (102;158)	134 (108;165)	142 (112;179)
Glycemic index 2	56 (3.9)	54 (4.0)	56 (3.4)	56 (3.4)	57 (3.5)	58 (3.8)
Ratio energy intake/basal metabolic rate 2	1.4 (0.4)	1.2 (0.3)	1.4 (0.4)	1.4 (0.4)	1.5 (0.4)	1.5 (0.4)
Misreporting of diet 3 ^,^ 5						
Under-reporters	27.5	44.3	28.4	25.7	21.0	18.1
Plausible reporters	67.4	54.3	68.2	69.5	72.6	72.3
Over-reporters	5.1	1.5	3.4	4.9	6.5	9.6

1 DED Dietary energy density based on solid foods only.

2 values are mean (SD).

3 values are percentages.

4 values are median (Q1, Q3).

5 Misreporting of diet was estimated by using the ratio of reported energy intake to the predicted basal metabolic rate (EI/BMR). Individuals with an EI/BMR<1.14 were defined as under-reporters, EI/BMR>1.14 and <2.1 as plausible reporters and EI/BMR>2.1 as over-reporters.

Of all sixteen food groups, differences in consumption of fruits explained most of the variation between DED in individuals (30%), followed by vegetables (12%), fats (11%) and, cakes and biscuits (6%) (**Table 2**). Of all macronutrients, saturated fat explained most of the variation in DED (20%), followed by mono- and disaccharide carbohydrates (9%) and plant protein (8%). DED was inversely associated with consumption of fruits and vegetables and intake of mono- and disaccharide carbohydrates, animal protein and plant protein but positively associated with consumption of fats, cakes and biscuits, and intake of saturated fat.

**Table 2:** Relationships of food groups and macronutrients with dietary energy density (DED; kcal/g) in the subcohort (n = 15,434): the EPIC-InterAct Study.

Food groups 2	Correlation with DED^3^	Linear relationship with DED^1^		
		β^4^	Partial R^2^ ^5^	Cumulative R^2^ ^6^
Fruits	−0.59	−0.09	0.30	0.30
Vegetables	−0.48	−0.12	0.12	0.42
Fats	0.32	0.57	0.11	0.53
Cakes and biscuits	0.27	0.19	0.06	0.58
Meat and meat products	0.23	0.11	0.03	0.61
Soups and bouillon	−0.16	−0.08	0.03	0.64
Potatoes and other tubers	0.11	0.03	0.02	0.66
Dairy (excl milk beverages)	−0.09	−0.04	0.02	0.68
Cereal and cereal products	0.21	0.05	0.02	0.70
Nuts and seeds	0.11	0.30	0.02	0.72
Sugar and confectionary	0.34	0.13	0.01	0.73

Macronutrients 7	Correlation with DED^3^	β^8^	Partial R^2^ ^5^	Cumulative R^2^ ^6^
Saturated fat	0.42	0.04	0.20	0.20
MOno- & disaccharides	−0.34	−0.02	0.09	0.29
Plant protein	−0.23	−0.02	0.08	0.37
Animal protein	−0.31	−0.07	0.06	0.43

1 Results obtained from stepwise linear regression analyses among the subcohort.

2 Sixteen food group variables (grams per day) were included: potatoes, vegetables, legumes, fruits, dairy products (except milk beverages), nuts and seeds, cereals, meat, fish, eggs, fats, sugar and confectionery, cakes and biscuits, condiments and sauces, soups, and miscellaneous.

3 Spearman's rank correlation coefficient presented which represent the correlation between DED and food groups or nutrients.

4 β regression coefficient represents the energy density (kcal/g) difference explained by 100 g foods.

5 Partial R^2^ (explained variance) represents the inter-individual variation in DED explained by the individual food group or nutrient. Only food or nutrient items had Partial R^2^>0.01 were listed here.

6 Cumulative R^2^ represents the sum of the inter-individual variation in DED explained by the specific food group or nutrient and previously listed food groups or nutrients.

7 Seven macronutrients (en% per day) were included: saturated fatty acids, monounsaturated fatty acids, polyunsaturated fatty acids, polysaccharides, mono-&disaccharides, animal protein and plant protein.

8 β regression coefficient represents the energy density (kcal/g) difference explained by 1% of the energy contributed by the individual nutrient.

Pooled HRs for T2D per 1 kcal/g increase in DED are presented (**Table** **3**). After adjustment for sex and age (model 1) an inverse association between DED and T2D was found, which became statistically significant following adjustment for known risk factors of diabetes (model 2, HR 0.88 (95% CI 0.79–0.99, *I* ^2^ = 23.0%). Thus an increase of 1 kcal/g in DED was associated with 12% lower hazard of T2D, which corresponds with a shift from the lowest to the highest quintile of DED. However, the inverse association disappeared after further adjustment for dietary misreporting (Model 3, HR 1.02 (95% CI 0.93–1.13; *I* ^2^ = 2.9%). HRs were also close to unity comparing quintiles of DED (data not shown).

**Table 3:** Pooled hazard ratios^1^ for the association between dietary energy density (DED; kcal/g) and incident type 2 diabetes in Europe: the EPIC-InterAct Study.

	Dietary energy density 2	
	HR (95% CI)	I^2^ ^3^
**Model**		
1: age sex	0.95 (0.86–1.06)	21.3
2: model 1 + risk factors DM^4^	0.88 (0.79–0.99)	23.0
3: model 2 + misreporting of diet^5^	1.02 (0.93–1.13)	2.9

BMR = basal metabolic rate; CI = confidence interval; DED = dietary energy density; DM = diabetes mellitus; EI = energy intake; HR = hazard ratio;

1 Analysis stratified by country and pooled using a random effect meta-analysis; based on 11,734 T2DM cases and 15,434 subcohort members (overlap n = 733).

2 Dietary energy density based on solid foods only.

3 I^2^ represents the variation in the estimate between countries attributable to heterogeneity.

4 Smoking status (current, never, former), physical activity (inactive, moderately inactive, moderately active, active), alcohol (g/day), energy intake from beverages (kcal).

5 Misreporting of diet was estimated by using the ratio of reported energy intake to the predicted basal metabolic rate (EI/BMR). Individuals with an EI/BMR<1.14 were defined as under-reporters, EI/BMR>1.14 and <2.1 as plausible reporters and EI/BMR>2.1 as over-reporters.

**Figure 1:**
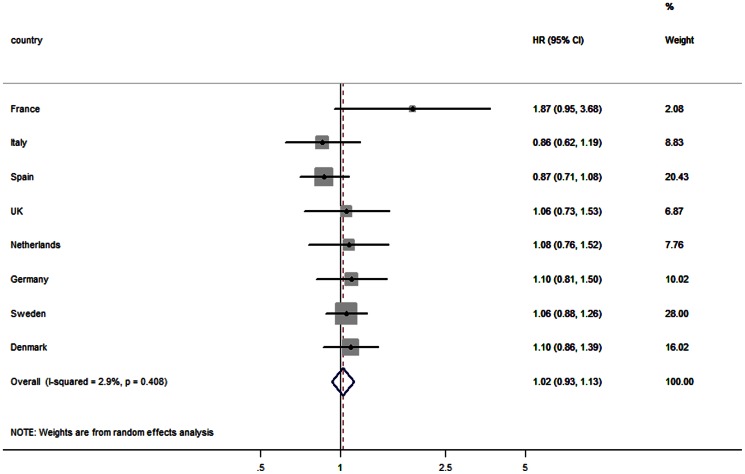
Association between dietary energy density and incident type 2 diabetes in Europe^1,2^. HR: hazard ratio per 1 kcal/g increase in energy density; 95% CI: 95% confidence interval for the HR. ^1^ Dietary energy density based on solid and semi-foods only. ^2^ Adjusted for age, sex, misreporting of diet (under-, plausible, over-reporter), smoking status (never, former, current), physical activity (inactive, moderate inactive, moderate active, active), alcohol (g/day), energy intake from beverages (kcal).

The association between DED and incidence of type 2 diabetes by country is shown in **Figure 1**. HRs are all not statistically significant, and showed weak positive associations in France, UK, the Netherlands Germany, Sweden and Denmark, whereas inverse associations were observed in Italy and Spain.

There was no evidence of interaction between DED and sex (p = 0.17), age (p = 0.41), BMI (p = 0.49), waist circumference (p = 0.63), physical activity (p = 0.96) or misreporting of diet (under- vs plausible reporters; p = 0.38).

**Figure 2:**
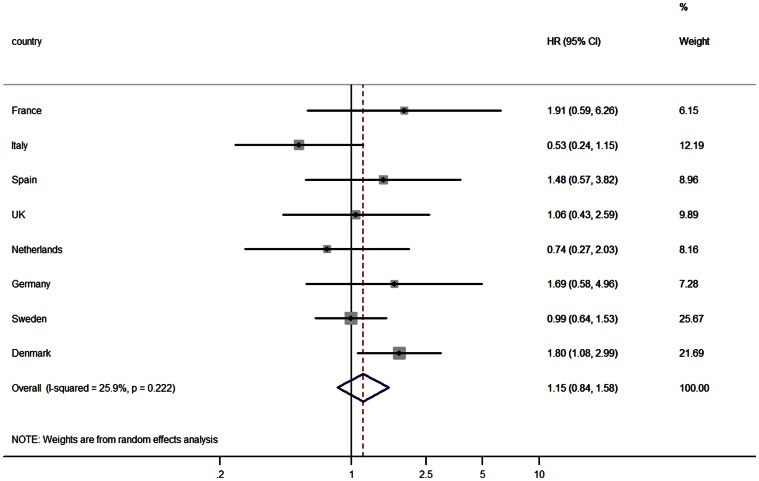
Association between dietary energy density and incident type 2 diabetes in Europe among plausible dietary reporters of energy with a normal body weight^1–3^. HR: hazard ratio per 1 kcal/g increase in energy density; 95% CI: 95% confidence interval for the HR. ^1^ Dietary energy density based on solid foods only. ^2^Adjusted for age, sex, smoking status (never, former, current), physical activity (inactive, moderate inactive, moderate active, active), alcohol (g/day), energy intake from beverages (kcal). ^3^ Normal weight is defined as a BMI<25 kg/m^2^ and plausible reporting of diet is defined as a ratio of energy intake versus estimated basal metabolic rate between 1.14 and 2.1.

The highest percentage of normal weight individuals (79.4%) and the lowest percentage of individuals classified as under-reporters of diet (9.5%) were observed in France (**Table 4**). The lowest percentage of normal weight individuals was observed in Spain (22.2%), whereas the highest percentage of under-reporters was observed in Germany (36.1%). Stratified analysis by misreporting of diet affected the HRs especially in individuals with a normal weight at baseline (**Table 5**). Among plausible dietary reporters with a normal body weight, a positive but not statistically significant association between DED and incident T2D was observed (HR 1.15, 95% CI (0.84–1.58) *I* ^2^ = 25.9; **Figure 2**)) whereas among those defined as dietary under-reporters DED tended to be inversely associated with incident T2D (HR 0.64, 95% CI (0.41–1.02). Among overweight and obese individuals, no clear differences in HRs between under- and plausible reporters were observed. Results from sensitivity analysis showed a statistically significant positive association between DED and incident T2D (HR 1.54 95% CI: 1.13–2.10, figure S1) when all foods and all beverages except water were included in the DED calculation. There was a large increase in heterogeneity (from 2.9% to 71.3%) when using this DED estimate.

**Table 4:** Characteristics of BMI status and misreporting of diet by country in the subcohort (n = 15,434): the EPIC-InterAct Study.

Country	N	% Women	BMI status (%)^1^			Misreporting of diet (%)^2^		
			Normal weight	Overweight	Obesity	Under-reporters	Plausible reporters	Over-reporters
France	549	100	79.4	15.7	4.9	9.5	78.3	12.2
Italy	1,921	66.9	46.8	39.6	13.7	16.9	71.9	11.2
Spain	3,457	61.7	22.2	48.8	29.0	29.1	66.4	4.5
UK	1,200	61.4	52.2	37.4	10.4	32.3	61.9	5.8
Netherlands	1,366	83.2	53.4	35.5	11.1	30.2	68.6	1.2
Germany	2,012	58.4	47.4	38.4	14.2	36.1	60.3	3.5
Sweden	2,852	57.1	53.7	35.6	10.7	29.0	67.0	4.1
Denmark	2,077	46.6	44.1	42.7	13.2	24.5	71.9	3.7

1 Normal weight was defined as a BMI<25 kg/m^2^, overweight as a BMI between 25 and 30 kg/m^2^ and obesity as a BMI ≥30 kg/m^2^.

2 Misreporting of diet was estimated by using the ratio of reported energy intake to the predicted basal metabolic rate (EI/BMR). Individuals with an EI/BMR<1.14 were defined as under-reporters, EI/BMR>1.14 and <2.1 as plausible reporters and EI/BMR>2.1 as over-reporters.

**Table 5:** Pooled hazard ratios^1^ for the association between dietary energy density and incident type 2 diabetes in Europe stratified by BMI status and misreporting of diet: the EPIC-InterAct Study^1^^–^ 3.

		BMI		
Misreporting of diet 4	HR (95% CI)	<25 kg/m^2^	25–30 kg/m^2^	≥30 kg/m^2^
		HR (95% CI)	HR (95% CI)	HR (95% CI)
Under-reporters	1.00 (0.85–1.18)	0.64 (0.41–1.02)	1.03 (0.79–1.35)	1.04 (0.72–1.48)
Plausible reporters	1.02 (0.88–1.19)	1.15 (0.84–1.58)	0.96 (0.78–1.19)	0.96 (0.74–1.24)
**HR (95% CI)** 5	1.01 (0.92–1.12)	0.98 (0.78–1.25)	0.97 (0.83–1.13)	0.93 (0.77–1.12)

BMI = body mass index; BMR = basal metabolic rate; CI = confidence interval; DED = dietary energy density; EI = energy intake; HR = hazard ratio.

1 Analysis stratified by country and pooled using a random effect meta-analysis; based on n = 11,045 T2DM cases and n = 14,162 subcohort members, (overlap n = 704).

Due small numbers no reliable estimates could be calculated for over-reporters (n = 1,220), and under-reporters in France (n = 88), which therefore are excluded from this analysis.

2 Dietary energy density based on solid foods only.

3 Adjusted for age, sex, smoking status (current, never, former), physical activity (inactive, moderately inactive, moderately active, active), alcohol (g/day), energy intake from beverages (kcal).

4 Misreporting of diet was estimated by using the ratio of reported energy intake to the predicted basal metabolic rate (EI/BMR). Individuals with an EI/BMR<1.14 were defined as under-reporters, EI/BMR>1.14 and <2.1 as plausible reporters and EI/BMR>2.1 as over-reporters.

5 Additionally adjusted for misreporting of diet.

## Discussion

Overall, this large European case-cohort study among 11,734 incident T2D cases and a subcohort of 15,434 participants showed no evidence for an association between DED of solid and semi-solid foods and risk of T2D. This observation was consistent across the eight participating countries, located in different geographical areas in Northern and Southern Europe.

The results of this study should be interpreted with caution because of the difficulties involved when assessing obesity related diet-disease relationships in epidemiological studies [31]. One of the limitations of this study is the use of DED estimates based on self-report of habitual food intake, which might have caused bias due to conscious or sub-conscious under- or over-reporting of specific food items [32]. In this study we observed large differences between countries in the number of participants classified as under-reporters. This could be due to the use of country-specific questionnaires. Also, the prevalence of overweight and obesity varied between countries. Under-reporting of energy intake has been shown to be more prevalent and severe among individuals with a higher body mass index [23], [33]. As obesity is a well-established risk factor for T2D the association between DED and T2D may be prone to bias due to obesity-related under-reporting. A recent analysis in EPIC showed that the BMI effect on under-reporting is the same across countries and that exclusion of individuals who misreported energy according to the Goldberg cut-off removed the obesity-related bias [34]. Results from our stratified analysis showed that among overweight and obese individuals HRs were close to unity for those categorized as under-reporters and those categorized as plausible reporters. However, in normal weight individuals categorized as under-reporters an inverse association between DED and risk of T2D was seen, whereas DED tended to be positively associated with incidence of T2D among normal weight individuals classified as plausible reporters. Risk of T2D was 15% higher per 1 kcal/g increase in DED, which corresponds to a change in DED from the lowest to the highest quintile. This association was not statistically significant, despite the large sample size in this sub group analysis. Overall, our results indicate that the obesity related underreporting did not affect our overall results much. Still, we cannot exclude an overall bias in energy reporting across all BMI categories.

The main strengths of this study are the large sample size, the large number of verified T2D cases, its prospective design, the variation in dietary intake across participants from eight European countries and the availability of information on important potential confounding variables such as smoking behavior, alcohol consumption, physical activity and measured waist circumference and body weight.

To our knowledge, the association between DED and risk of T2D has been investigated only once, by Wang et al. [14]. DED was calculated using FFQs and included all beverages except water. They reported a 20% higher risk of diabetes per unit (kJ) increase in DED in the EPIC-Norfolk study (n = 21,919) which included 725 incident T2D cases (HR 1.20 (95% CI 1.05–1.37). This positive association is in line with the result of our sensitivity analysis in which we used the same DED calculation method.

So far, no clear consensus has been reached on the calculation of DED. A previous review of the literature identified eight different calculation methods which mostly differed in the inclusion or exclusion of water, other energy-free beverages and energy-containing beverages [22]. In the current study, data on consumption of bottled and tap water was not available in all centres. Because detailed water intake is generally not collected in epidemiologic studies, excluding other energy-free beverages such as coffee and tea eliminates potential bias that could be created by excluding only water [22]. However, coffee and tea often provide energy through added sugar or milk and this should be taken into account. Unfortunately, in our study (as in many others) no such information was available. Independent of the limitations mentioned above, we decided *a priori* to include only solid and semi-solid foods and to exclude caloric and non-caloric beverages from the DED calculation while partially adjusting for energy intake from beverages in the models. The rationale for this choice was, amongst others (see Methods section) that inclusion of beverages into DED calculation is associated with higher day to day variance within individuals. This may lead to biased associations when examining health outcomes [22]. This is supported by the result of our sensitivity analysis in which DED was calculated including all food and beverages except water. The pooled estimates showed a large increase in heterogeneity (I^2^ = 71.3%) compared to our main results (I^2^ = 2.9%).This means that the association between DED and risk of T2D is less consistent across countries when drinks are included into the DED calculation. To compare, an I^2^ of 25%, 50% and 75% could roughly be interpreted as indicating low, medium and high heterogeneity [35]. Furthermore, when individual study results are inconsistent (i.e. heterogeneity is considerable), the obtained pooled estimate is less valid [35], [36]. Together, this favors the exclusion of drinks from DED calculation and adjusting models for the energy intake from beverages. On the other hand, it could be speculated that, despite the methodological limitations, the observed higher T2D risk when drinks are included in the DED calculation is driven by a positive association between energy-containing beverages and risk of T2D as reported in literature [37], [38]. This would suggest that energy density of drinks rather than the energy density of solid foods is important in determining the risk of diabetes.

It has been hypothesized that diets high in DED affect risk of T2D indirectly via an increase in body fat mass. Literature shows that foods with a higher energy density are more palatable and less satiating as compared to foods with a lower energy density and can thus lead to passive over consumption and a higher energy intake [39], [40]. Moreover, prospective studies have shown that DED is positively associated with risk of (abdominal) obesity, a well-established risk factor of T2D [8], [9], [10].

On the other hand, it can be postulated that high DED diets have a direct effect on T2D risk. In the current study, a diet high in DED is characterized by a lower intake of fiber, fruit and vegetables, a higher intake of energy and saturated fat and a higher glycemic index (GI) and glycemic load. This is in agreement with findings of previous studies [6], [10], [41]. Saturated fat has been suggested to adversely affect insulin sensitivity of muscles as well as glucose stimulated insulin secretion [12]. High GI diets can rapidly increase postprandial glucose concentrations. This may lead to pancreatic exhaustion as a result of the increased demand for insulin [42]. In addition, high GI diets can increase postprandial free fatty acid release, directly increasing insulin resistance [13].

The composition of low energy dense diets meet the dietary recommendations given by WHO to promote human health and to prevent diet-related chronic diseases [43]. In addition, high energy-dense diets have been found to predict (abdominal) obesity [8], [9], . Therefore, despite the fact that there currently is no conclusive evidence for an association between DED and risk of T2D, choosing low energy dense foods should be promoted as they support the current dietary recommendations.

In conclusion, the results of this large European case-cohort study do not provide evidence for an association between DED of solid and semi-solid foods and the risk of T2D. However, we found some indication that misreporting of diet and BMI status may have obscured a positive association between DED and risk of T2D, and that such an association, if any, would most likely be small.

## Supporting Information

Figure S1 **Association between dietary energy density and incident type 2 diabetes in Europe^1,2^.** HR: hazard ratio per 1 kcal/g increase in energy density; 95% CI: 95% confidence interval for the HR. ^1^ Dietary energy density based on all foods and beverages (except water). ^2^ Adjusted for age, sex, misreporting of diet (under-, plausible, over-reporter), smoking status (never, former, current), physical activity (inactive, moderate inactive, moderate active, active), alcohol (g/day).
(TIF)
