# Supplementary figures and images for: The Association between Dietary Energy Density and Type 2 Diabetes in Europe: Results from the EPIC-InterAct Study
Source: PLoS One. 2013 May 16;8(5):e59947. doi: 10.1371/journal.pone.0059947 (PMC3655954; doi:10.1371/journal.pone.0059947)

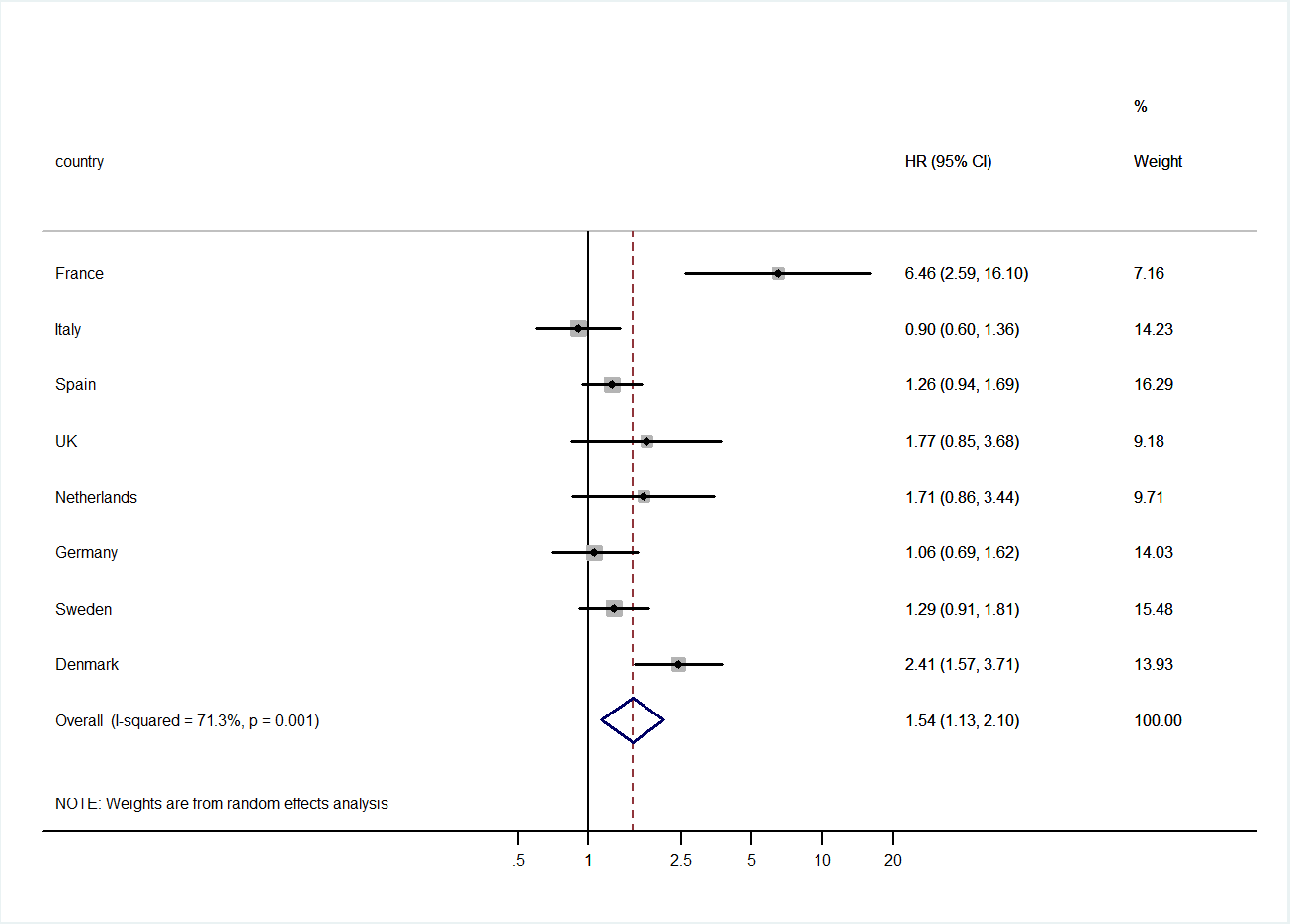

Supplement: Figure S1 — Association between dietary energy density and incident type 2 diabetes in Europe1,2. HR: hazard ratio per 1 kcal/g increase in energy density; 95% CI: 95% confidence interval for the HR. 1 Dietary energy density based on all foods and beverages (except water). 2 Adjusted for age, sex, misreporting of diet (under-, plausible, over-reporter), smoking status (never, former, current), physical activity (inactive, moderate inactive, moderate active, active), alcohol (g/day). (TIF) [file pone.0059947.s001.tif]
